# Production of high-quality two-dimensional gel electrophoresis profile for marine medaka samples by using Trizol-based protein extraction approaches

**DOI:** 10.1186/s12953-020-00161-9

**Published:** 2020-05-02

**Authors:** Celia Sze-Nga Kwok, Kaze King-Yip Lai, Sai-Wo Lam, Kin-Ka Chan, Steven Jing-Liang Xu, Fred Wang-Fat Lee

**Affiliations:** Department of Science, School of Science and Technology, The Open University of Hong Kong, Hong Kong, SAR China

**Keywords:** Protein extraction, Two-dimensional gel electrophoresis, Trizol, Medaka, Proteomics

## Abstract

**Background:**

Marine medaka is among the most popular models of fish species for ecotoxicology and environmental research and proteomic studies are useful tools for understanding the molecular responses of medaka upon exposure to different environmental stressors. The preparation of high-quality protein samples is the key to producing high-quality two-dimensional gel electrophoresis (2-DE) results for proteomic analysis. In recent years, Trizol-based protein extraction has been gaining popularity because of its promising performance in producing high-quality 2-DE as well as the convenience of the method.

**Methods:**

Three Trizol-based approaches (Trizol method, Aliquot Trizol method and Trizol method with a commercial clean-up kit) were used to extract proteins from a marine medaka sample and 2-DE profiles were produced. Quality of the 2-DE profiles and effectiveness of the extraction methods were evaluated. For comparison, two common protein extraction methods (lysis buffer method and trichloroacetic acid (TCA)/acetone precipitation extraction) were also applied in parallel to Trizol-based approaches.

**Results:**

Any of the three Trizol-based approaches produced a high-quality 2-DE profile of marine medaka compared with both lysis buffer method and TCA/acetone precipitation extraction. In addition, Trizol method with a commercial clean-up kit produced the best 2-DE profile in terms of background clarity, number of spots and resolution of proteins.

**Conclusions:**

Trizol-based approaches offered better choices than traditional protein extraction methods for 2-DE analysis of marine medaka. The modified version of Trizol method with a commercial clean-up kit was shown to produce the best 2-DE profile.

## Background

Marine medaka is a popular fish model for toxicological research mainly because of its small size, short gestation period and ability to tolerate wide ranges of salinity and temperature. As many toxicants disrupt cellular functions by altering gene expression, comparative analyses of gene expression profiles are frequently done in mechanistic toxicology [1–3]. This could be done by obtaining the protein profiles of the whole fish, suspected target organs or even cell lines under normal and intoxicated conditions. When there is little information on the organs affected, one may start with the whole fish before organ-specific profiling. Conventionally, in comparative proteomics, proteins in a complex mixture are first separated by two-dimensional gel electrophoresis (2-DE) so that differentially expressed proteins can be identified by mass spectrometry (MS) [4]. In the past decade, a considerable amount of attention has been given to gel-free approaches, which has overcome many limitations of gel-based methods, such as laboriousness, low throughput, poor reproducibility and incomplete protein coverage. However, some irreplaceable values of gel-based methods can complement the weaknesses of gel-free methods. One example is that identification of protein isoforms and posttranslational modifications is usually not feasible in gel-free analysis due to the high percentage of amino acid sequence homology, whereas said sequences could be differentiated in 2-DE as isoforms [5–7]. Also, modified proteins are likely to have different isoelectric points or molecular weights. Furthermore, 2-DE would allow de novo sequencing analysis on specific protein spots that were visualized on the gel. This is particularly useful for the proteomic study of non-model organisms [8]. Thus, 2-DE remains a useful tool for comparative protein profiling [9–12].

Cells and tissue usually contain interfering compounds, which mainly interfere with the first dimension of 2-DE and induce severe streaking of the gel. This phenomenon substantially reduces the number of distinctly resolved spots on the 2D gel. To improve the quality of 2-DE profiles, different protein extraction methods were developed by scientists. In 2002, researchers developed a complicated protein preparation method, which included tedious procedures such as protease inhibition, nuclease treatments and desalting steps [13]. This method was time consuming and resulted in the loss of protein spots. A few years later, others supported the use of urea/Triton X-100 extraction with TCA/acetone precipitation for protein preparation to reveal a higher yield of proteins, a larger number of protein spots and a clearer background compared with other methods at the cost of additional time for the preparation of buffers and solvents [14]. Recently, there is growing evidence that high-quality 2-DE profiles could be successfully produced with the use of Trizol reagent and notably, many of them were from marine samples (Table 1). Compared to traditional protein extraction methods, Trizol-based method is labor-saving as it does not require additional nucleases to remove nucleic acid [15, 40] or extra procedures to remove excess salts [13, 18]. Membrane proteins can be effectively extracted [41] and solubility of proteins is desirable [42]. Besides, Trizol method offers simultaneous extraction of DNA, RNA and proteins, which saves both samples and time for comprehensive analysis of genomes, transcriptomes and proteomes [42]. Despite the high potential of Trizol method, some problems were still found in Trizol-extracted samples. In 2009, a group of researchers reported that the intensity of protein spots obtained from Trizol-extracted samples was often vague [14]. A study later reported that streaking was frequently observed in the background of 2-DE profiles, probably due to the incomplete removal of interfering substances from Trizol-extracted samples [43]. Since interfering substances may vary with different sample types, trials on more types of samples would help explore the potential usage of Trizol in protein extraction. Also, modifications of the conventional method could be tested to obtain better results.

**Table 1:** Previous reports of using Trizol protein extraction method for 2-DE production

Published Year	Sample Type	References
2006	Halophilic archaea	[15]
2007	Human neck squamous cell carcinoma cells Rat spinal cord tissue	[16] [17]
2008	Dinoflagellates Human heart tissue	[18] [19]
2009	Human breast cancer cells Mites	[20] [21]
2011	Dinoflagellates Human kidney carcinoma cells Leguminous plant	[22] [23] [24]
2012	Caudal gland tissue Dinoflagellates Human neuroblastoma cells	[25] [26, 27] [28]
2013	Dinoflagellates *Haliotis* Human non-cancerous liver tissue Mediterranean mussel; Olive flounder; Polychaetes	[29] [30] [31] [32]
2015	Dinoflagellates *Haliotis* Heart biopsies	[33, 34] [35] [36]
2018	Dried seafood and dried tonic food Reef corals	[37] [38]
2019	Adipose-derived stem cells	[39]

None of the 2-DE studies on marine medaka has adopted a Trizol method in their protein extraction steps, except a recent study that used Coomassie blue staining [44]. Although the resulting gel showed little background noise, this might not be the case when silver staining, which is at least 10 times more sensitive than Coomassie blue staining, is used [45]. Therefore, the success of the use of a Trizol-based method to produce high-quality 2-DE for medaka samples remains unclear. In this study, we aimed to examine the suitability of various protein extraction methods for future applications of medaka in toxicological studies using 2-DE. An aliquot element and a follow-up commercial clean-up kit were incorporated to classical Trizol method with the prospect of improvement in results. Two commonly used extraction methods, (lysis buffer method and TCA/acetone precipitation) were also included for comparison. The performance of each method was evaluated and compared in several aspects including number of protein spots, background signal, resolution as well as protein yield. Furthermore, a preliminary comparative proteomic analysis of medaka exposed to a toxic microalgae was conducted using the most suitable extraction method so as to demonstrate its applicability in medaka toxicological studies.

## Methods

### Materials

All the chemicals and solvents used were of analytical grade and were purchased from Sigma–Aldrich Corporation (USA), unless otherwise stated. Medaka fish were obtained from State Key Laboratory of Marine Pollution, City University of Hong Kong.

### Preparation of medaka samples

The fish were cultivated at 30 ± 1 ppt, which is the middle of its range of salinity tolerance [46, 47]. All fish tanks were maintained at 25 °C with a 12:12 h light/dark cycle and constant aeration [48]. Adult medaka (more than 3 months old) were picked and killed by placing in ice slurry before protein extraction.

### Protein extraction

**Fig. 1:**
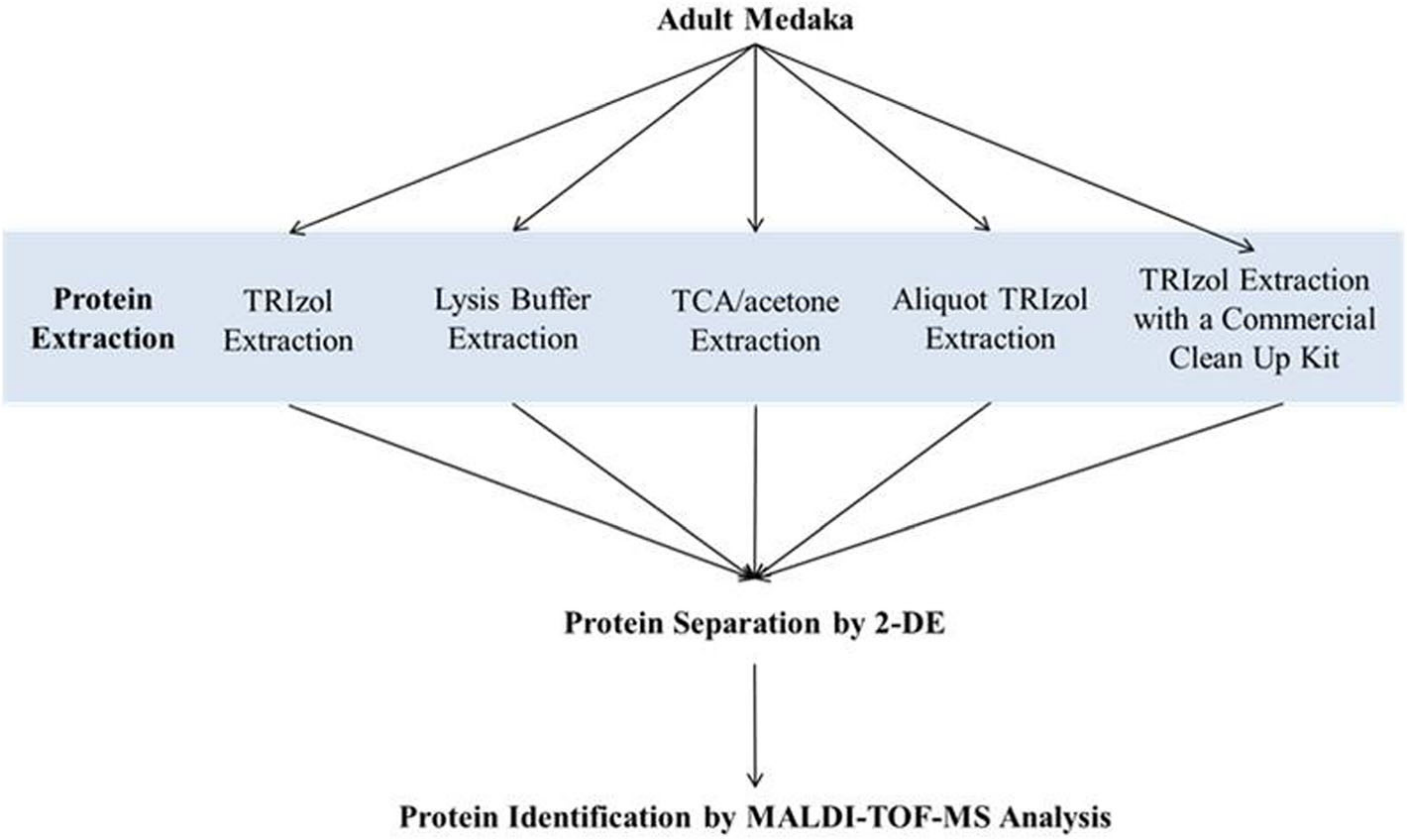
A flow chart of experimental design. Five extraction methods were selected in this study

Five extraction methods were selected in the present study, including Trizol method, lysis buffer method, TCA/acetone precipitation, Aliquot Trizol method and Trizol method with a commercial clean-up kit (Fig. 1). All reagents and buffer solutions used were maintained ice-cold throughout the whole extraction process. In each extraction method, one to two whole adult fish were weighed and then mixed with the reagent or buffer in triplicate for homogenization.

### Trizol method

Trizol reagent (Life Technologies, USA) was used according to the manufacturer’s instructions with some modifications, as mentioned in Lee’s study [18]. One milliliter of Trizol reagent was added to a whole medaka followed by sonication on ice for at least 15 min with a pulse of 20 s (amplitude: 90%). The debris was removed by centrifugation at 1500 g for 15 min at 4 °C and 200 μL of chloroform was added to the supernatant and shaken vigorously for 15 s. The sample was incubated at room temperature for at least 15 min and spun at 12,000 g for 15 min at 4 °C. The upper colorless layer and the precipitate between the two layers were discarded and 300 μL of ethanol was added and mixed well. The mixture was then centrifuged at 6000 g for 5 min. The supernatant was transferred to a new 2 mL microcentrifuge tube and mixed with isopropanol. The mixture was incubated at room temperature for at least 1 h for protein precipitation. Then, the precipitate was washed twice using ethanol. Finally, 50 μL of lysis buffer (7 M urea, 2 M thiourea, 4% CHAPS, 40 mM Tris, pH 8.5) was added to solubilize the protein pellet. The solubilized protein was stored at − 80 °C until use.

### Lysis buffer method

One milliliter of lysis buffer was added to a whole medaka followed by sonication on ice for at least 15 min with a pulse of 20 s (amplitude: 90%). After sonication, the sample was centrifuged at 12,000 g for 15 min at 4 °C. The supernatant was collected in a new microcentrifuge tube and stored at − 80 °C until use while the pellet was discarded.

### TCA/acetone precipitation

One milliliter of lysis buffer was added to a medaka sample followed by sonication on ice for at least 15 min with a pulse of 20 s (amplitude: 90%). After centrifugation, the supernatant was transferred to a new microcentrifuge tube and 3 times the volume of ice-cooled 10% TCA/acetone solvent was added for precipitation. The sample was then incubated at − 20 °C overnight. After the overnight incubation, the sample was centrifuged at 12,000 g for 15 min at 4 °C. The supernatant was discarded to obtain the pellet, which was washed three times with ice-cooled acetone. The pellet was then air-dried for a short duration (1–2 min) and 50 μL of lysis buffer was added for the solubilization of the protein pellet. The solubilized protein was stored at − 80 °C until use.

### Aliquot Trizol method

Five hundred microliters of Trizol reagent was added to a whole medaka. Then, the mixture was sonicated with the time and amplitude mentioned previously in Trizol method. Instead of being kept in a single tube in conventional Trizol method, the sonicated product was evenly distributed in five new microcentrifuge tubes, with 100 μL each. Each tube was then topped up to 1 mL with Trizol reagent, after which the procedures for Trizol method continued as mentioned previously. After washing the protein precipitate with ethanol, 10 μL of lysis buffer was added to solubilize the protein pellet in each tube. Extracted proteins from these five aliquots were combined and stored at − 80 °C until use.

### Trizol method with a commercial clean-up kit

Proteins from medaka were first extracted using Trizol method as mentioned previously and completely dissolved in the lysis buffer. After that, the solubilized protein was further purified using a commercial 2-D clean-up kit (GE Healthcare, USA). Standard procedures from the user manual of the kits were followed. Briefly, three times the volume of the precipitant was added to the sample and mixed well by vortex. The sample was then incubated on ice for at least 15 min. Then, three times the volume of the coprecipitant was added to the sample and mixed well by vortex. The protein was precipitated and collected by centrifuging the microcentrifuge tube at 12,000 g for 10 min. Next, the supernatant was removed. After that, three to four times the pellet volume of coprecipitant was added. The microcentrifuge tube was centrifuged at 12,000 g for 5 min. The supernatant was removed and discarded. A small volume of 18 mΩ water was added on top to cover the pellet. Then, 1 mL of wash buffer (prechilled at − 20 °C for at least 1 h) and 5 μL of wash additives were added to the pellet and mixed well by vortex. The microcentrifuge tube was incubated at − 20 °C for at least 1 h and continually mixed by a vortex every 10 to 15 min. The sample was centrifuged at 12,000 g for another 10 min, after which the supernatant was removed and discarded. The remaining pellet was air-dried for a short duration (1–2 min). Finally, 50 μL of lysis buffer was added to solubilize the protein pellet. The solubilized protein was stored at − 80 °C until use.

### Protein quantification, two-dimensional gel electrophoresis and imaging analysis

Protein concentration was measured using the Bradford protein assay (Bio-Rad). This acidic dye was added to the protein solution and absorbance of 595 nm was determined using a microplate reader. Standard curve was constructed by using bovine serum albumin (BSA) as the protein standard. The amount of extracted protein was quantified using a modified Bradford protein assay (Bio-Rad, USA) [49].

A total of 100 μg protein extracted from the sample was added to rehydration buffer (7 M urea, 2 M thiourea, 4% CHAPS, 0.2% dithiothreitol (DTT), 1% immobilized pH gradient (IPG) buffer pH 3–10) before rehydration of a pH 4–7 IPG strip. In the first dimension of the 2-DE, each IPG strip was rehydrated with 340 μL of the loaded rehydration buffer for 16 h. Isoelectric focusing (IEF) was performed by using a Protean IEF cell (Bio-Rad, USA). The voltage program was set as follows: 1 h at 100 V, 2 h at 300 V, 2 h at 1000 V, 2 h at 4000 V and 5 h at 8000 V. After the IEF process, the strip was equilibrated with an equilibration buffer (50 mM Tris, pH 8.8, 6 M urea, 30% glycerol, 2% sodium dodecyl sulphate, 1% DTT and trace amounts of bromophenol blue) for 30 min. After the equilibration, the strip was transferred to another equilibration buffer (containing 1% iodoacetamide) and incubated again for 30 min in the dark at room temperature. The IPG strip was then rinsed with running buffer (25 mM Tris, 192 mM glycine, 0.2% sodium dodecyl sulphate, pH 8.3) and placed onto a freshly-cast 2D gel (13 cm × 15 cm). During the second dimension of the 2-DE, proteins in the IPG strip were moved to and separated by 10% polyacrylamide gel at 15 mA per gel at room temperature. For protein visualization and further analysis, the gel was stained with silver nitrate according to the procedures performed by Blum et al. [50]. The stained gel was scanned using a Gel Doc XR system (Bio-Rad, USA) with automatic selection of the best exposure time and the captured gel image was saved for record. For qualitative and quantitative examination of protein spots on the gel image, Melanie 7 (GeneBio, Switzerland) was used according to the user manual.

### Exposure of medaka to a toxic microalgal culture

A preliminary ichthyotoxicity study of a toxic dinoflagellate *Karenia mikimotoi* was conducted. The strain of *K. mikimotoi* was isolated from *K. mikimotoi* blooms in Yim Tin Tsai from December 2015 to February 2016. The identity of the cells was confirmed based on their morphological features. A monoclonal culture has been successfully established and the culture is currently maintained in L1 medium. The algal culture was maintained at 22 °C under a 12:12 h light: dark cycle with a light intensity of ~ 3000 Lux. Twenty medaka fish were exposed to 2.5 × 10^4^ cells/mL of *K. mikimotoi* in a fish tank containing algal culture medium (L1 medium); a duplicate set-up without *K. mikimotoi* cells was used as a control. Artificial seawater used for medium preparation was filtered through a 0.45 μm nitrocellulose membrane (Whatman) and autoclaved before use. Dissolved oxygen was maintained at a minimum level of 5–7 mg/L. Feeding of medaka was ceased 24 h prior to toxicity tests. The exposure lasted for 24 h and the mortality and symptoms of medaka was recorded. Pooled samples of medaka fish from both treatment group and control group were collected respectively after 20 mins of exposure for comparative proteomic analysis.

### In-gel digestion

Analysis of 2-DE profiles using Melanie 7 was performed according to the user manual. The gel plugs (around 1 mm^3^ each) containing the interested protein spots were excised from the silver-stained gel. The silver-stained gel was first destained by adding destaining solution that contained 0.01 g/mL potassium ferricyanide and 0.016 g/mL sodium thiosulfate. After that, the gel plug was washed twice with 25 mM ammonium bicarbonate (NH_4_HCO_3_) for 5 min each. Next, the gel plug was washed with 25 mM NH_4_HCO_3_ in 50% acetonitrile (ACN) for 5 min. Finally, the colorless gel plug was dehydrated by adding 100% ACN. The dried gel pieces were reduced by incubation with 10 mM DTT at 55 °C for 45 min and then alkylated by incubation with 10 mM iodoacetamide at room temperature in the dark for 45 min. Further, the gel plug was washed with 25 mM NH_4_HCO_3_ in 50% ACN and dried again with 25 mM NH_4_HCO_3_ in 100% ACN. In total, 3 μL of freshly prepared trypsin (20 mg/mL; Promega, USA) solution was added onto the dried gel pieces. After a 30 min incubation on ice, the remaining trypsin was removed to minimize the amount of the digested trypsin in the sample. Trypsin digestion was performed overnight at 37 °C. Then, digested peptides inside the gel were eluted with 0.1% trifluoroacetic acid (TFA) in 50% ACN with the aid of ultrasonication. The eluted peptide solution was finally dried with a Labconco CentriVap DNA Vacuum Concentrator and stored at − 80 °C until further use.

### MALDI-TOF-MS analysis

Firstly, 1 μL of saturated α-cyano-4-hydroxycinnamic acid (2 mg/mL) in 0.1% TFA with ACN (2:1) was coated on each spot of the anchor-chip target plate (Bruker, Germany). The dried peptides were resuspended with 2 μL of 0.1% TFA with ACN (2:1) and then 1 μL was added onto the dried anchor spots. After the spot dried, the anchor spot was briefly washed with 0.1% TFA and subsequently recrystallized with 1 μL of recrystallization solution (ethanol: acetone: 0.1% TFA = 6:3:1). The mass spectra, ranging from 700 to 3000 Da, were determined using the reflector mode of Bruker Autoflex III Series High-Performance MALDI-TOF & TOF-TOF systems after calibration with an external peptide calibration standard (Bruker, Germany) was conducted. Spectra from 500 shots at different positions on the target plates were combined to generate a peptide-mass fingerprint. The obtained peptide masses were searched against the National Center for Biotechnology Information (NCBI) protein database of medaka (NCBI:txid8090) by using the MASCOT search engine [51, 52]. The selected database consisted of 38,099 sequences and 22,982,484 residues on 12 Oct 2015.

## Results and discussion

### Methodological comparison of various protein extraction methods

Trizol reagent is a ready-to-use reagent that was designed to isolate RNA, DNA and proteins from a single sample of cells or tissue from humans, animals, plants, yeasts, or bacteria [4, 53, 54]. The procedures detailed in the manual were easy to follow and it took approximately 3 h to complete the extraction process (Table 2). In related studies, Trizol method has been applied in the protein extraction of several algal species [41, 44]. Our team has also demonstrated recently the potential of using Trizol-based extraction methods in proteomic study of dried seafood [37].

**Table 2:** Comparison of solvents and kits required, total time consumed and ease of handling in five protein extraction methods

Protein Extraction Method	Solvents and Kits Required	Total Time Consumed	Ease of Handling
Trizol method	Trizol reagent, chloroform, ethanol, isopropanol and lysis buffer	3 h	Easy to follow the procedures on manual
Lysis buffer method	Lysis buffer	0.5 h	With only a few steps Solvent Preparation is needed prior to extraction
TCA/acetone precipitation	10% TCA in acetone, cold acetone, DTT and lysis buffer	Overnight^a^ (12–20 h)	Solvent Preparation is needed prior to extraction
Aliquot Trizol method	Trizol reagent, chloroform, ethanol, isopropanol and lysis buffer	4 h	Handling multiple samples simultaneously
Trizol method with a commercial clean-up kit	Trizol reagent, chloroform, ethanol, isopropanol, 2-D clean-up kit, Mili-Q water and lysis buffer	6 h	Many steps in the clean-up procedure

^a^TCA/acetone precipitation used in this study was an overnight method. A normal TCA/acetone precipitation protocol could be completed within a few hours

During sample preparation, there are four essential steps: (1) cell disruption, (2) inactivation of proteases, (3) removal of interfering substances and (4) protein solubilization [55]. The most critical step is removing as many interfering substances as possible during protein extraction procedures. Both lysis buffer method and TCA/aceteone precipitation have been commonly used to prepare various sample types, such as mammals (mice), marine organisms (mussels) and dinoflagellates [56–58]. Moreover, they have been widely applied to prepare fish samples, such as medaka and zebrafish [59–64]. Consequently, conventional extraction methods, including lysis buffer method and TCA/acetone precipitation, were applied on medaka for comparison. Lysis buffer method consisted of only a few steps and could be completed in approximately 30 min, whereas TCA/acetone precipitation was conducted overnight (at least 12 h) to ensure complete precipitation (Table 2).

In the working procedures of aliquot Trizol method, both the total volume of Trizol solution used and the number of extractions increased. The removal capacity was enhanced at least 5-fold by aliquoting a single tube of medaka tissue sample into five tubes for Trizol method. Similarly, the commercial clean-up kit (GE Healthcare, USA) was originally designed for samples resulting in poor 2-DE profiles due to high conductivity, high salt levels, interfering substances, or a low sample concentration. With the aid of the clean-up kit, proteins are precipitated extensively while the interfering substances such as detergents, salts, lipids and phenol and nucleic acids remain in the solution. This kit has four components: a precipitant, coprecipitant, wash buffer and wash additive. Precipitant renders protein insoluble, whereas a coprecipitant can coprecipitate with proteins and enhance their removal from the solution. A wash buffer is used to remove nonprotein contaminants from protein precipitates and wash additives promote the rapid and complete resuspension of the proteins. The kit can also help scale up larger volumes or more dilute samples. Additionally, the presence of salts or charged detergents can affect first-dimension electrophoresis. These interfering substances can be removed by the kit. Because the processing times of these two methods are approximately 4 to 6 h (Table 2), lengthy procedure time seems to be the major drawback regarding the use of aliquot Trizol method and Trizol method with a commercial clean-up kit.

### Overall patterns of 2-DE profiles generated from different extraction methods

**Fig. 2:**
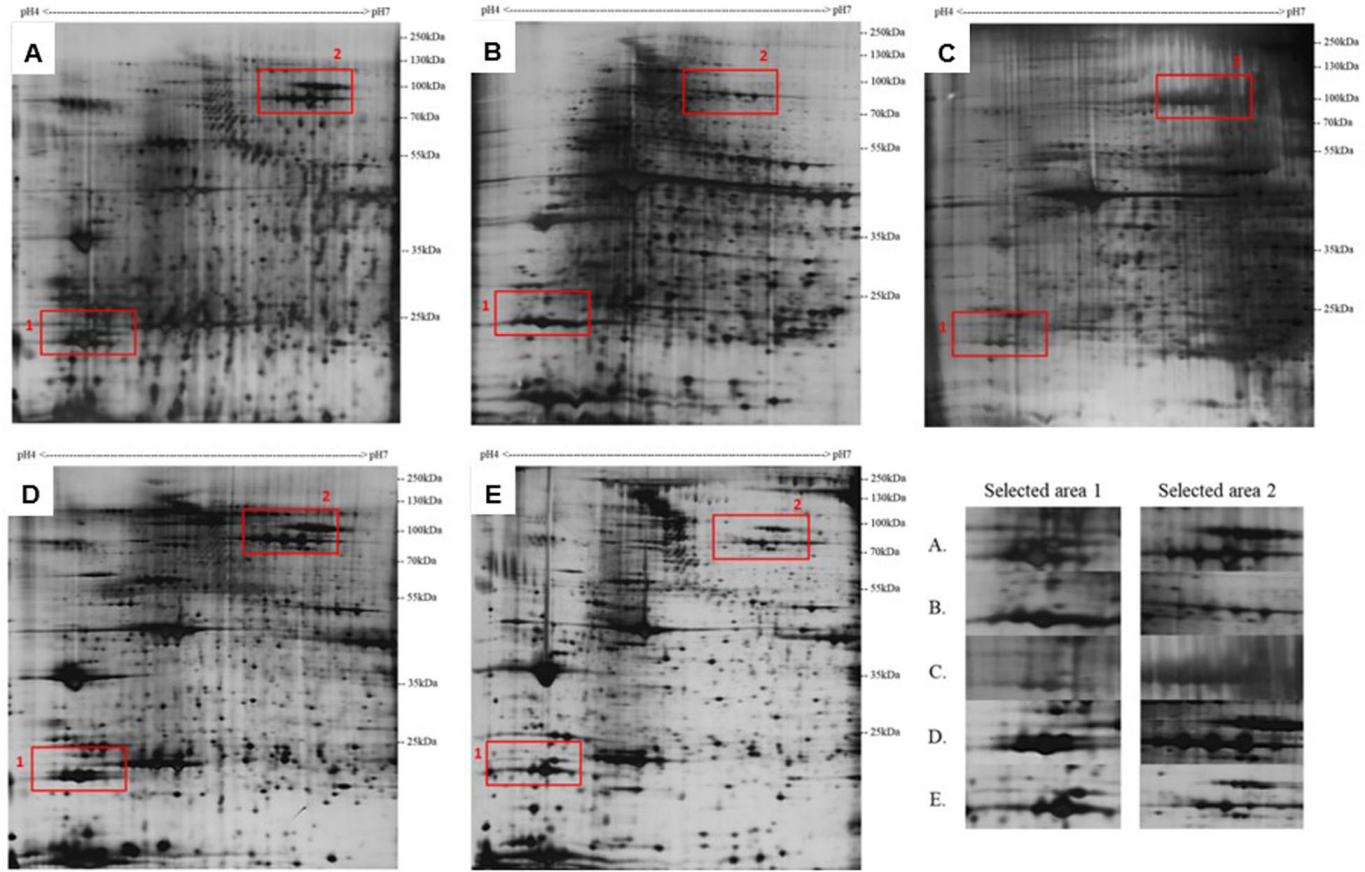
2-DE profiles (pH 4–7) of protein samples from medaka. Proteins were extracted using **a** Trizol method, **b** lysis buffer method, **c** TCA/acetone precipitation, **d** aliquot Trizol method and **e**. Trizol method with a commercial clean-up kit. A total of 100 μL of each protein extract was loaded onto the strips and silver staining was used. Magnified images of selected areas on the 2-DE profile of each method are shown at the right bottom corner

Obtaining high-quality 2-DE protein profiles is the most important step in a comparative proteomic study. As shown on the 2-DE protein profile generated from lysis buffer method (Fig. 2b), protein loss probably occurred near four corners. This profile had a high level of background and streaking. Such poor quality and resolution would greatly impede the gels from 2-DE comparative analyses. For the 2-DE protein profile generated from TCA/acetone precipitation, the resolution was even worse than the one from lysis buffer method (Fig. 2c). These problems might be due to the method’s inability to remove interfering substances. Some unwanted substances might also be precipitated and result in the poor resolution of protein spots and a low-quality 2-DE protein profile. Some researchers have achieved favorable outcomes using these two extraction methods, probably because they usually worked on dissected parts of medaka instead of a whole fish. For example, Tian’s group performed 2-DE analysis on the gills and brains of medaka after they were exposed to an algal toxin (brevetoxin-1). Proteins were extracted from the medaka samples using TCA/acetone precipitation [60]. Another group conducted 2-DE analysis on the liver of medaka after exposure to inorganic mercury by using TCA/acetone for protein extraction [64]. However, occasionally, it might be necessary to examine a whole fish to provide a more comprehensive understanding of in vivo molecular responses. Therefore, a whole fish sample was used in the present study to determine whether Trizol – based methods could be a suitable protein extraction approach for proteomic studies on medaka.

2-DE protein profile generated from Trizol-extracted proteins of medaka had high background noise; in addition, some of the protein spots were not well separated (Fig. 2a). This profile was not satisfactory for comparative studies, although it is much better than that of the lysis buffer method and TCA/acetone precipitation. It demonstrated that certain interfering substances from medaka samples may not be effectively removed by classical Trizol method. We hypothesized that the incomplete removal of interfering substances may be attributed to the amount of interfering substances in the sample exceeding the maximum removal capacity of Trizol reagent. Therefore, the use of aliquot Trizol method might help to solve this problem. Modification methods, including a pre-Trizol method (aliquot Trizol method) and post-Trizol method (Trizol method with a commercial clean-up kit), were evaluated. As noted previously, the types and amounts of interfering substances are sample dependent and there is no single protein extraction method that can be universally applied to all types of samples for the generation of 2-DE. Therefore, modification of existing methods is commonly applied in the method optimization. Here, the overall 2-DE profiles using the two modified Trizol methods were notably better (Fig. 2).

### Numbers of protein spots on 2-DE profiles generated from different extraction methods

**Fig. 3:**
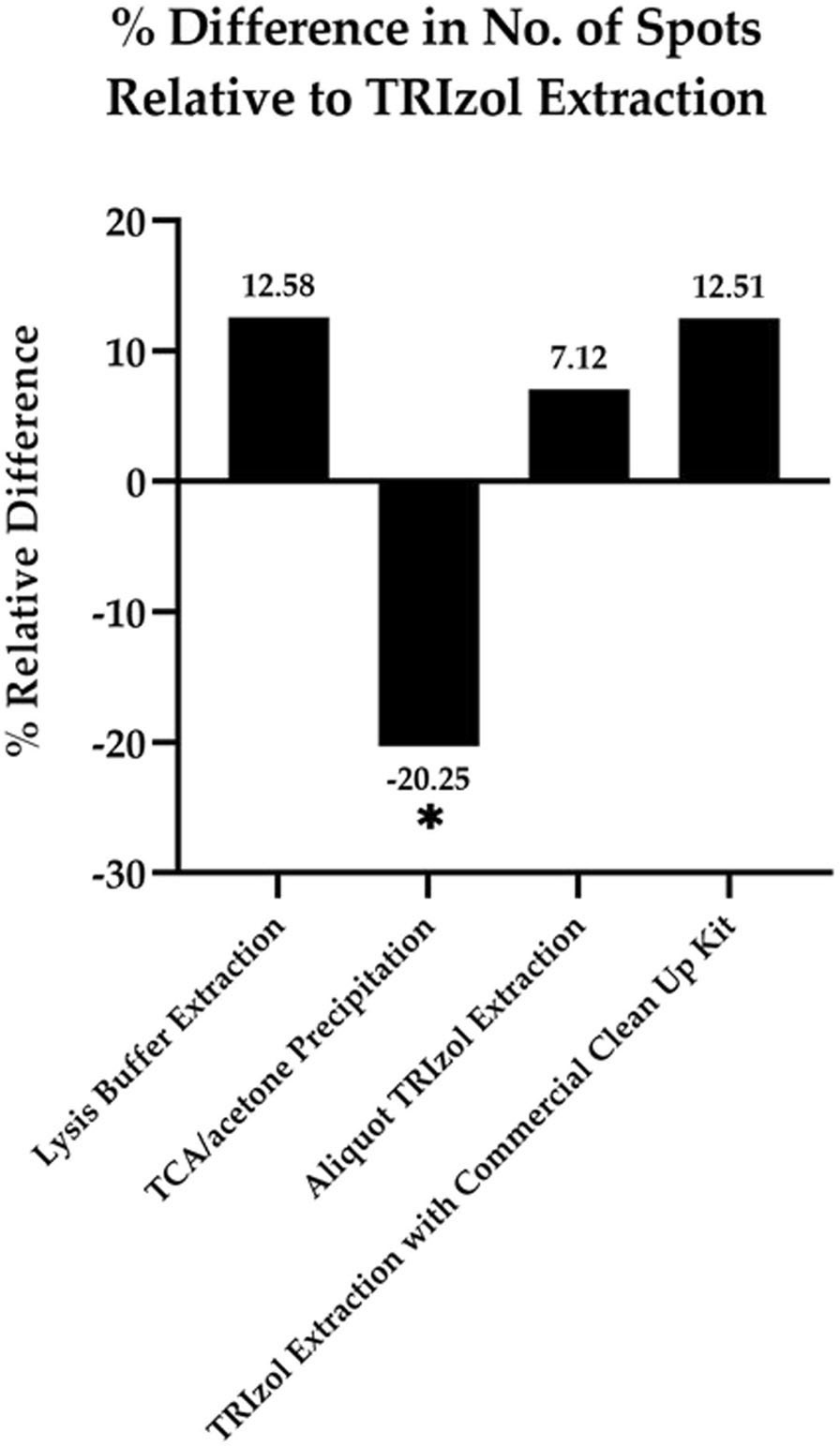
Percentage difference in number of protein spots on the 2-DE profiles of proteins in medaka between Trizol method and other extraction methods. Each protein sample was extracted from five medaka fish, with 100 μL of protein loaded onto the strip. * indicates a significant difference (P ≤ 0.05) compared with Trizol method (Tukey’s HSD method)

Because this study focused on validating and improving the performance of Trizol-based approaches, the classical Trizol method acted as the pillar against which four other extraction methods (two non-Trizol-based methods and two modified Trizol-based methods) could be compared to. Although the number of spots from lysis buffer method was approximately 13% higher than that of Trizol method (Fig. 3), the background quality was very poor and many protein spots were masked by the noise signal (Fig. 2b). In TCA/acetone precipitation method, the number of spots was approximately 20% less than that of Trizol method and was the lowest among all extraction methods (Fig. 3, Table 3). The relatively low-quality 2-DE protein profile generated from this method was accompanied by high background noise and serious streaking (Fig. 2c). Thus, some protein spots could not be successfully determined using the 2-DE analyzing software (Melanie 7, GeneBio).

**Table 3:** Comparison of 2-DE profiles of proteins in medaka extracted using five methods in terms of No. of protein spots, quality of background and resolution of proteins. Each protein sample was extracted from five medaka fish, with 100 μL of protein loaded on the strip. The value inside the bracket is the standard deviation of three independent trials in each protein extraction method. “+++” = good, “++” = average, “+” = fair and “-” = poor

Protein Extraction Method	No. of Protein Spots	Quality of Background	Resolution of Proteins
Trizol method	1447 (32.47)	+	+
Lysis buffer method	1629 (112.59)	–	+
TCA/acetone precipitation	1154 (96.91)*	–	–
Aliquot Trizol method	1550 (65.91)	++	++
Trizol method with a commercial clean-up kit	1628 (40.28)	+++	+++

*Statistically significant difference (*P* ≤ 0.05) compared with Trizol method (Tukey’s HSD method)

By contrast, in aliquot Trizol method, the quality of the background and resolution of protein spots were considerably improved; additionally, the number of spots was approximately 7% higher than that for Trizol method approach (Fig. 3, Table 3). The intensities and patterns of protein spots shown in the magnified images of the two selected areas of the 2D gel appeared to be similar to those from Trizol method (Fig. 2). The number of spots on the 2-DE protein profiles from Trizol method with a commercial clean-up kit was almost 13% higher than that from Trizol method alone and was comparable to the largest spot number, which was achieved using lysis buffer method (Fig. 3). The appearance of extra spots might be attributed to the increase in protein solubility resulting from the removal of interference substances that could not be effectively removed by Trizol method.

### Protein yields in different extraction methods

**Fig. 4:**
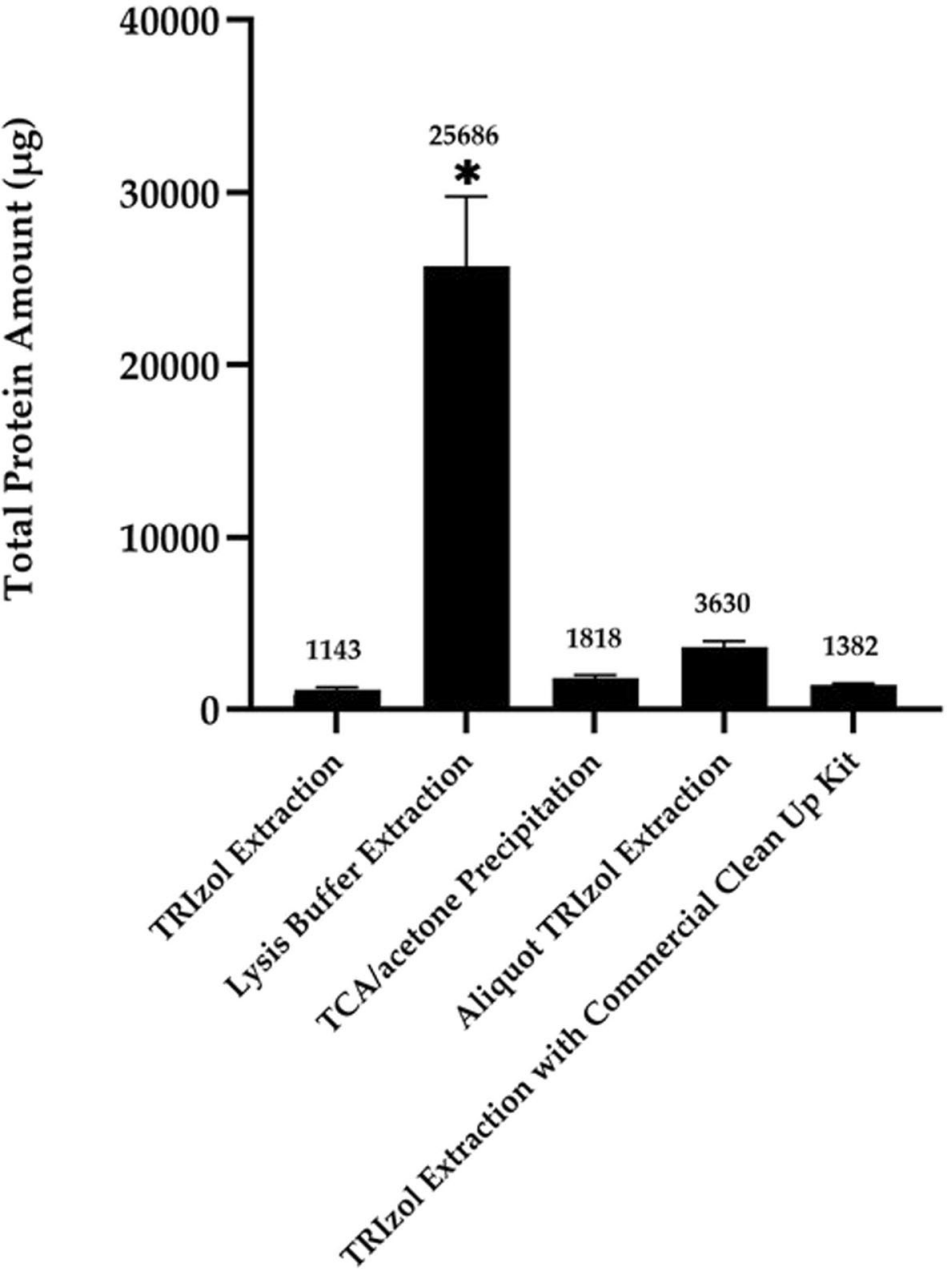
Yield of extracted proteins from five extraction methods. The mean values are displayed above the bars. Each sample was standardized as a 1 g medaka sample. * indicates a significant difference (*P* ≤ 0.05) compared with Trizol method (Tukey’s HSD method)

Protein yield is an important criterion in protein extraction. In the present study, to compare the yield of each extraction method, each sample was standardized into a 1 g medaka sample. Compared to Trizol method, protein yield in lysis buffer method was significantly higher (up to 22-fold) (*P* ≤ 0.05; Fig. 4). No significant difference (P ≤ 0.05) was noted among the other four extraction methods. A possible reason for this was the protein loss that occurred from the repeated phase separation of aqueous and organic layers in these four methods. For example, a small portion of proteins might be lost into an upper aqueous phase while most were partitioned in a lower organic phase in Trizol method. For TCA/acetone precipitation, although the procedures were conducted overnight, certain amount of proteins may not be well precipitated. For aliquot Trizol method, the increase of protein yield may attribute to the increased volume of Trizol solution used. This could enhance the capacity for protein solubilisation. For another modified Trizol method, extracted proteins were cleaned and concentrated using a commercial clean-up kit. Thus, the protein yield with the aid of a clean-up kit was slightly higher than that from conventional Trizol method.

When solely focusing the yield of extracted proteins, lysis buffer method completely outplayed the other four methods with 26 mg protein yield per gram of medaka sample (Fig. 4). However, it is not regarded as a good extraction method for comparative 2-DE study since the background noise was too prominent and the quality of 2-DE profile was far from satisfactory. It should be noted that 2-DE quality remains as the most important factor. On the other hands, protein yields of the three Trizol-based methods were all over 1 mg per gram of sample (Trizol method: 1.14 mg; Aliquot Trizol method: 3.63 mg; Trizol method with a commercial clean-up kit: 1.38 mg). On average, the weight of an adult medaka is around 0.7 g [65] while the optimal loading amount of proteins to a 17 cm IEF strip is usually up to 130 μg when using silver staining [66]. This implies that the protein yields of Trizol-based methods were far more than enough for several trials. Moreover, the protein yield in Trizol method with a clean-up kit was also comparable to that in the commonly used TCA/acetone precipitation method (Fig. 4). Considering all the factors examined, Trizol method with a commercial clean-up kit performed better than others in terms of quality of the background, resolution of separated proteins, number of protein spots, protein yield and spot intensity. Therefore, applying Trizol method with a commercial clean-up kit is recommended 2-DE studies of medaka samples.

### Differentially expressed proteins in medaka after exposure to K. mikimotoi

**Fig. 5:**
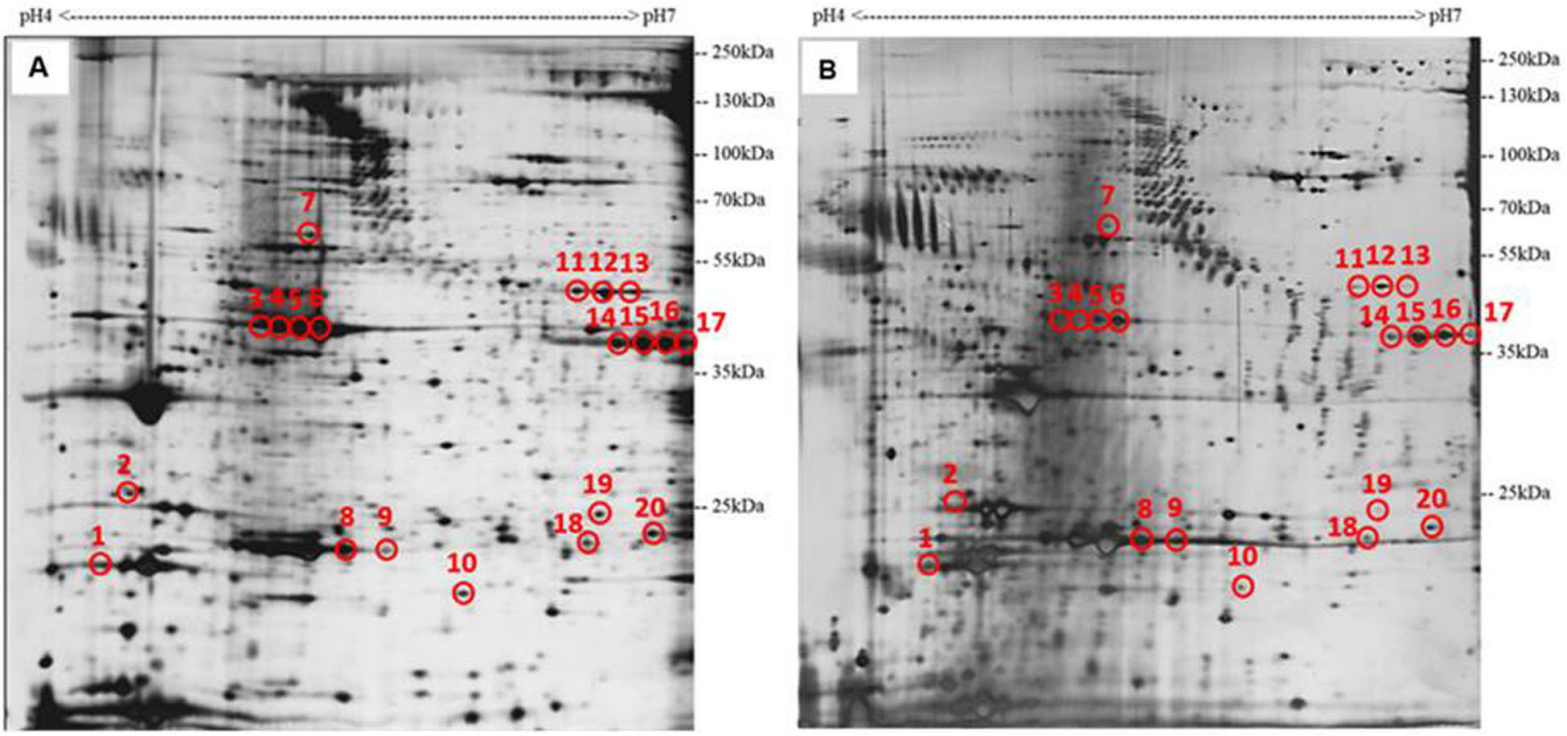
2-DE profiles of (**a)** untreated medaka and (**b)** medaka exposed to *K. mikimotoi*. Proteins were extracted using Trizol method with a clean-up kit. The loading amount of proteins was 100 μg and silver staining was used. Differentially expressed protein spots were circled

The ultimate step of a 2-DE analysis is to identify separated protein spots by using MS. Therefore, it is necessary to verify the success of this crucial step for any extraction method before applications [28]. Peptide mass fingerprinting (PMF) is a common approach for protein identification in MALDI-TOF MS. Protein was digested into peptides using trypsin, after which each peptide mass was generated to form a “fingerprint” by MALDI-TOF-MS. The PMF spectrum was then be searched against the peptide mass fingerprint database [67, 68]. In this study, we conducted preliminary experiments using 2-DE to analyse the medaka after exposure to *Karenia mikimotoi* cells. *K. mikimotoi* is a well-known species of fish-killing microalgae. Blooms dominated by this species are often associated with massive fish and shellfish kills around the world. However, the fish killing mechanism of this species is still remains unclear. Unveiling the molecular responses of medaka upon exposure to *K. mikimotoi* would provide important insights and ultimately aid in understanding the possible molecular mechanisms of algal ichthyotoxicity in fish. From the preliminary toxicity tests, we found that the mortality of marine medaka upon exposure to *K. mikimotoi* cells was 100% within 60 min. The response was acute and the shortest fish-killing time was around 25 min. Symptoms such as loss of balance and gasping for air were observed in the medaka after 10 min of exposure. No mortality and symptoms were observed in the control group throughout the exposure experiments. Certain critical parameters including temperature, pH, dissolved oxygen (DO), salinity and concentration of ammonia were constantly monitored and maintained at an acceptable level throughout the exposure period. Proteins were extracted from the pooled samples of treated and non-treated medaka collected at 20 mins after the exposure to the algal cells using Trizol method with a commercial clean-up kit and separated by 2-DE. The gel images of the treatment group were compared to that of the control group and differentially expressed proteins were identified. Twenty resolved protein spots that exhibited significant differences (with at least 2-fold change) were picked from the 2-DE profiles and subjected to MS analysis (Fig. 5). Nineteen out of twenty protein spots were shown to be down-regulated in response to *K. mikimotoi* exposure and all of them were successfully identified from the Swiss Prot protein sequence database of medaka. The information about the identified proteins were summarized in Table 4.

**Table 4:** Identities of differentially expressed proteins extracted from medaka after exposure to *K. mikimotoi* using Trizol method with a commercial clean-up kit. MASCOT search engine was used for PMF analysis. Protein scores higher than 58 were significant (*p* ≤ 0.05)

Spot	pI	MW (kDa)	Protein Name	Accession Number	Mascot Score	Sequence Coverage (%)	Fold Change^a^
1	4.64	20	Myosin light chain 1, skeletal muscle isoform	gi|432,932,023	71	50	−2.0
2	4.66	27	14–3-3 protein beta/alpha-1-like	gi|432,959,056	73	39	−3.0
3	5.23	42	Muscle actin OlMA1	gi|1,552,222	85	39	−3.7
4	5.23	42	Muscle actin OlMA1	gi|1,552,222	110	40	−8.6
5	5.23	42	Muscle actin OlMA1	gi|1,552,222	115	37	− 9.8
6	5.23	42	Muscle actin OlMA1	gi|1,552,222	79	33	−10.4
7	5.15	58	Keratin, type II cytoskeletal 8-like isoform X1	gi|432,864,501	109	33	−3.5
8	5.66	30	Apolipoprotein A-I	gi|327,358,583	117	52	−9.2
9	5.66	30	Apolipoprotein A-I	gi|327,358,583	85	42	2.2
10	5.97	23	Beta-crystallin A1–1	gi|432,890,713	83	47	−2.1
11	6.17	47	Beta-enolase	gi|432,957,740	129	52	−2.8
12	6.17	47	Beta-enolase	gi|432,957,740	160	58	−3.5
13	6.17	47	Beta-enolase	gi|432,957,740	119	46	−3.2
14	6.32	42	Creatine kinase M-type	gi|765,137,894	92	31	−5.2
15	6.32	42	Creatine kinase M-type	gi|765,137,894	97	31	−9.6
16	6.32	42	Creatine kinase M-type	gi|765,137,894	99	29	−6.3
17	6.32	42	Creatine kinase M-type	gi|765,137,894	88	35	−3.1
18	6.09	23	Beta-crystallin A2 isoform X2	gi|432,964,694	99	55	−3.5
19	6.59	27	Beta-crystallin B1	gi|432,884,641	92	52	−3.8
20	6.90	26	Triosephosphate isomerase	gi|432,908,784	79	39	−2.1

^a^A negative value denotes down-regulation after treatment

Among the identified protein spots, myosin light chain (spot 1) and keratin (spot 7) are closely linked to the oxidative stress response of medaka, which reflected the toxic symptoms developed during ichthyotoxicity tests [59, 69]. For example, metabolic response might reflect agonal respiration. Muscle response includes loss of balance and body twitching (spasms). Oxidative stress is usually related to an imbalance between the generation and elimination of reactive oxygen species (ROS) [59, 69, 70]. 14–3-3 protein (spot 2) was suggested to play critical roles in cell cycle control through binding with signaling molecules [71]. Muscle actin OlMA1 (spots 3–6) is a skeletal muscle actin expressed in somatic and head muscles and was found to be related to ATP binding [70]. Apolipoprotein A-I (spots 8–9) plays important roles in lipid transportation and metabolism and is the key protein component in high-density lipoprotein in plasma [72]. Beta-enolase (spots 11–13), also named enolase 3, is an abundant protein in striated muscle tissue commonly known as a heat-labile allergen in fish [73, 74]. Creatine kinase (spots 14–17) and triosephosphate isomerase (spot 20) were closely linked to metabolic response of medaka [59, 69]. Spots 10, 18 and 19 were identified as beta-crystallin proteins, which are transparent and found in eye lens [75]. These results support the feasibility and applicability of protein identification and comparative proteomic studies using Trizol method with a commercial clean-up kit.

## Conclusions

Trizol-based methods offered several advantages. It is a fast and simple method. More importantly, this method offers simultaneously extraction of RNA, DNA and protein in one go from the same sample. In this study, three Trizol – based methods were compared against two commonly used methods (lysis buffer method and TCA/acetone precipitation) for the generation of 2-DE from medaka sample. Our data demonstrated that Trizol-based methods generally produced 2-DE results with better quality compared with lysis buffer method and TCA/acetone precipitation. However, when using conventional Trizol method, substantial amounts of background images and streaking were observed on the gel, which greatly impede it from comparative proteomic analysis. Therefore, modified methods namely a pre-Trizol method (aliquot Trizol method) and post-Trizol method (Trizol method with a commercial clean-up kit) were evaluated. Both modifications significantly improved the quality. In particular, the Trizol with clean-up kit approach further purified the sample after the basic treatment of Trizol and produced the best overall 2-DE images of medaka proteins. These findings have demonstrated the promising potential of Trizol-based extraction approaches, especially Trizol method with a commercial clean-up kit, for gel-based proteomic analyses of medaka samples.

## Data Availability

All data generated and analyzed during this study are included in this published article.

## Abbreviations

TCA: Trichloroacetic acid
2-DE: Two-dimensional gel electrophoresis
MS: Mass spectrometry
2D-DIGE: Two-dimensional difference gel electrophoresis
DTT: Dithiothreitol
IPG: Immobilized pH gradient
IEF: Isoelectric focusing
ACN: Acetonitrile
TFA: Trifluoroacetic acid
